# Effect of continuous planting on *Casuarina equisetifolia* rhizosphere soil physicochemical indexes, microbial functional diversity and metabolites

**DOI:** 10.3389/fpls.2023.1288444

**Published:** 2023-12-14

**Authors:** Yuhua Wang, Jianjuan Li, Mingzhe Li, Xiaoli Jia, Yuhong Cai, Mingyue Hu, Qingxu Zhang, Pengyuan Cheng, Shaoxiong Lin, Wenxiong Lin, Haibin Wang, Zeyan Wu

**Affiliations:** ^1^ College of JunCao Science and Ecology, Fujian Agriculture and Forestry University, Fuzhou, China; ^2^ Fujian Provincial Key Laboratory of Agroecological Processing and Safety Monitoring, College of Life Sciences, Fujian Agriculture and Forestry University, Fuzhou, China; ^3^ Editorial Department, Fujian Academy of Forestry Survey and Planning, Fuzhou, China; ^4^ College of Life Science, Longyan University, Longyan, China; ^5^ College of Tea and Food, Wuyi University, Wuyishan, China

**Keywords:** *Casuarina equisetifolia*, rhizosphere soil, long-term monoculture plantations, microbial functional diversity, soil metabolomics, soil nutrient

## Abstract

Continuous planting has a severe impact on the growth of *Casuarina equisetifolia*. In this study, the effects of three different long-term monocultures (one, two and three replanting) on the physicochemical indexes, microbial functional diversity, and soil metabolomics were analyzed in *C. equisetifolia* rhizosphere soil. The results showed that rhizosphere soil organic matter content, cation exchange capacity, total and available nitrogen, total and available phosphorus, and total and available potassium contents significantly decreased with the increasing number of continuous plantings. The evaluation of microbial functional diversity revealed a reduction in the number of soil microorganisms that rely on carbohydrates for carbon sources and an increase in soil microorganisms that used phenolic acid, carboxylic acid, fatty acid, and amines as carbon sources. Soil metabolomics analysis showed a significant decrease in soil carbohydrate content and a significant accumulation of autotoxic acid, amine, and lipid in the *C. equisetifolia* rhizosphere soil. Consequently, the growth of *C. equisetifolia* could hinder total nutrient content and their availability. Thus, valuable insights for managing the cultivation of *C. equisetifolia* and soil remediation were provided.

## 1 Introduction

Continuous planting refers to the recurrent monoculture of the same plant in the same soil, and long-term continuous planting may lead to the deterioration of the soil environment, which in turn inhibits the growth of plants (Hufnagel et al., 2020; Tan et al., 2021). *Casuarina equisetifolia* is an evergreen tree of Casuarinaceae, native to Oceania, the Pacific Islands and Southeast Asia. It was introduced to China in the 1950s, and is now mainly distributed in the coastal areas of Guangdong, Fujian and Hainan provinces of China (Zhong et al., 2010). *C. equisetifolia* has a lifespan of about 30 years, reaches a height of 30 meters and a maximum diameter at breast height of 70 centimeters, and can be harvested after 10 years of planting. The wood of *C. equisetifolia* is solid and economically important as a ship’s sole and as a building material. Secondly, *C. equisetifolia* has a well-developed root system and strong resistance, which plays an important role in soil and water conservation and tidal erosion prevention in the coastal zone, and has been widely planted in the coastal areas of China (Athikalam and Karur Vaideeswaran, 2022). Due to the more specialized environment of the coastal sandy areas, fewer species are suitable for planting, so *C. equisetifolia*, which has long been the main species planted, can only continue to be replanted *in situ* after harvesting (Anbarashan et al., 2022). *C. equisetifolia* was reported to have decreased in height, diameter and volume by about 23.7%, 24.4% and 29.0%, respectively, after three continuous plantings (Zhang et al., 2000). The emergence of this phenomenon of continuous planting obstacle greatly constrains the sustainable development of *C. equisetifolia*’s protective forest resources.

There have been numerous reports on the effect of continuous planting on soil microbial ecosystems, with studies focusing on herbal medicines, agricultural crops, vegetable (Li et al., 2021a; Pang et al., 2021; Wu et al., 2021). However, there is still less information on the effects on soil microorganisms after continuous planting of artificial timber trees, especially *C. equisetifolia* (Liu et al., 2020; Li et al., 2021b; Xu et al., 2021; Ma et al., 2023). Previous studies, using high-throughput sequencing, analysed the effect of continuous planting on the microbial diversity of *C. equisetifolia* rhizosphere soil and found a significant decrement in the microbial population, especially probiotics, and diversity after continuous planting, whereas the pathogenic bacteria significantly increased, and the nutrient cycling capacity of the soil was impeded (Zhou et al., 2019). The microbial diversity and gene expression related to nitrogen cycling in the soil were further analysed, showing a reduction in the nitrogen cycling capacity and a decrease in the available nitrogen content in the soil subject to *C. equisetifolia* recurrent monoculture (Zhou et al., 2021; Zhou et al., 2022). This series of studies found that continuous planting leads to a reduction in the nutrient cycling capacity of the soil from a soil microbial perspective. The plant root system is an important tissue for sensing changes in the soil environment. Plant root systems have been reported to influence the number, species, and function of rhizosphere soil microorganisms through the release of root secretions when sensing changes in the external environment, thereby altering the nutrient cycling of the rhizosphere soil in order to adapt to the environment (Shen and Lin, 2021; Tan et al., 2022; Ye et al., 2023). Therefore, in-depth analysis of the effects of continuous planting of *C. equisetifolia* on rhizosphere soil microorganisms and nutrient cycling from the perspective of rhizosphere soil metabolites is of great significance in further revealing its growth and changes in the rhizosphere ecosystem.

Rhizosphere soil metabolomics is an integrated study of low molecular weight compounds in plant rhizosphere soils and is an important tool for evaluating the diversity of soil microbial communities and soil function (Withers et al., 2020). In recent years, metabolomics technology was applied to analyze the interactions among metabolites, microorganisms, and environmental factors, and their impacts on soil function (Brown et al., 2021; Liang et al., 2021; Sun et al., 2022; Wu et al., 2022). However, studies using soil metabolomics to analyze the effects of metabolites in rhizosphere soils of continuous planting *C. equisetifolia* on microorganism functional diversity and nutrient cycling have rarely been reported. An in-depth understanding of the effects of continuous planting on soil metabolites and microbial functions in the root system of *C. equisetifolia* and their interactions is important for clarifying how continuous planting leads to changes in soil metabolites in the root system of *C. equisetifolia*, which in turn affects the functions of the microbial community and alters the nutrient cycling of the soil.

Accordingly, in this study, *C. equisetifolia* rhizosphere soils were collected from the first, second, and third continuous plantings to analyze the effects of continuous plantings on nutrient cycling, microbial functional diversity, and metabolites in the rhizosphere soils. Soil metabolomics analysis was used to screen and obtain metabolites for key changes in the rhizosphere soil of continuous planted *C. equisetifolia* and to analyze the effects of soil metabolites on microbial function and soil nutrient cycling. From the perspective of rhizosphere soil microorganisms and metabolites, this study initially revealed the causes of growth retardation of *C. equisetifolia* due to continuous planting, with a view to providing certain references for the cultivation and management of continuous planting of *C. equisetifolia*.

## 2 Materials and methods

### 2.1 Experimental site and rhizosphere soil sample collection

The researches of this study were performed at the national protective forest farm (118°55′ E, 24°35′ N), an area of about 433 ha located in Chihu Township, Hui’an County, Fujian Province, China. Climate conditions were those typical of the southern subtropical climatic zone of China, with an annual mean temperature of 19.8°C and an annual rainfall of 1,029 mm. *C. equisetifolia* plants were grown on sandy soil, with a stand density of about 950 plants·ha^-1^ and managed in accordance with the “Technical regulation on cultivation of casuarina seedlings and trees” (LY/T 3092-2019) issued by the State Forestry and Grassland Administration of the People’s Republic of China (National Forestry and Grassland Administration of the People's Republic of China, 2019).

In March 2018, *C. equisetifolia* seedlings were planted in plots that had never cropped with *C. equisetifolia*. These plots were defined as M1 (first planting). Plots cultivated with *C. equisetifolia* from 1987 to 2018 in which *C. equisetifolia* plants were cut, removed and re-planted in 2018 were selected to define the M2 (second planting) experimental thesis. Plots in which *C. equisetifolia* was planted in 1987, cut and replanted in 2011, removed and replanted in March 2018, defined M3 (third planting) experiment. All plots were transplanted with 2-year-old seedlings (0.8 m in height and 0.9 cm in diameter). M1, M2, and M3 were set up for three replicate plots, each with an area of 30 × 30 m (950 trees·ha^-1^, i.e., 85 plants per replicate).

In March 2022, the rhizosphere soils of *C. equisetifolia* with different number of continuous plantings (M1, M2, and M3) were collected for the determination of soil physicochemical indexes, functional diversity of soil microorganisms, and soil metabolites. Rhizosphere soil was sampled as follows: 6 plants were randomly selected by the “S” sampling method. The leaf litter layer was removed, and the upper layer of soil was shoveled out up to the root system (about 30 cm). The fine roots were cut, and the rhizosphere soil was removed by gently shaking (with a small brush), collected and mixed well into a self-sealing bag. A total of about 300 g was considered as one replicate. The collected rhizosphere soil was placed in an ice box. For each experimental plot, three independent replicates were set up.

### 2.2 Rhizosphere soil characterization

#### 2.2.1 Physicochemical indexes

Rhizosphere soil samples were naturally air-dried, ground, and passed through a 2 mm sieve before physicochemical indexes determination. Soil pH, organic matter content (OM), cation exchange capacity (CEC), total nitrogen (TN), total phosphorus (TP), total potassium (TK), available nitrogen (AN), available phosphorus (AP) and available potassium (AK) were determined with the methods described by Lu (2000). Briefly, soil pH was determined by the water leaching potential method (2.5:1 of water-soil ratio); OM was determined by oxidation with potassium dichromate; CEC was determined by ammonium acetate exchange; TN was determined by Kjeldahl nitrogen fixation; AN was determined by alkaline dissolution diffusion; TP and AP were determined by molybdenum antimony colorimetric assay; and TK and AK were determined by flame atomic absorption spectrophotometry.

#### 2.2.2 Microbial functional diversity

The functional diversity of rhizosphere soil microorganisms of *C. equisetifolia* was determined using the BIOLOG ECO microplate method (Zhang et al., 2023). Briefly, 10 g of freshly collected rhizosphere soil sample was taken in a conical flask containing 90 mL of sterile saline, sealed and placed in a shaker, shaken at 120 r·min^-1^ for 10 min, and left to stand for 3 min. 5 mL of supernatant was removed and 45 mL of sterile water was added to obtain a 1:100 dilution, which was repeated once to obtain a 1:1000 dilution. The diluted solution was added to the BIOLOG ECO microplate by adding 150 μL to each well, and the well with 150 μL of sterile water was added as a blank control, and the absorbance was immediately measured at 590 nm using an enzyme labeling apparatus and recorded as the initial absorbance. The BIOLOG ECO microplates were incubated at 28°C in the dark and the absorbance at 590 nm was measured regularly for 7 consecutive days. The functional diversity of the soil microbial community was expressed as average well color development (AWCD) per well on BIOLOG ECO microplates. AWCD was calculated as [∑(C-R)]·31^-1^, where C is the absorbance measured in each of the 31 wells and R is the absorbance measured in the control well. Based on the types of the 31 carbon sources in the BIOLOG ECO microplate, the carbon sources were categorized as carbohydrate, carboxylic acid, phenolic acid, fatty acid, amines and amino acid.

#### 2.2.3 Metabolomics

Soil metabolites were extracted with reference to the method of Ye et al. (2023). Briefly stated, fresh collected rhizosphere soil was weighed 0.5 g, added to 1 mL of methanol:isopropanol:water (3:3:2 V/V/V) extract, vortexed for 3 min, ultrasonicated for 20 min, and centrifuged for 3 min at 12,000 r·min^-1^ at 4°C. The supernatant was transferred to a sample vial, 0.02 mL of internal standard (10 μg·mL^-1^) was added, and nitrogen was blown dry for further sample derivatization. Sample derivatization was performed by mixing the sample with 0.1 mL of methoxyamine pyridine (15 mg·mL^-1^) and incubating at 37°C for 2 h. 0.1 mL of N,O-bis(trimethylsilyl)trifluoroacetamide (containing 1% trimethylchlorosilane) was added, and the sample was vortexed and shaken at 37°C for 30 min. After derivatization, 0.2 mL of the liquid was taken and diluted to 1 mL by adding n-hexane, passed through a 0.22 μm filter tip and tested by GC-MS.

Agilent 8890 gas chromatograph coupled to a 5977B mass spectrometer with a DB-5MS column (30 m length × 0.25 mm i.d. × 0.25 μm film thickness, J&W Scientific, USA) was employed for GC-MS analysis of the extracting solution. Helium was used as carrier gas, at a flow rate of 1.2 mL·min^-1^. Injections were made in the front inlet mode with a split ratio 5:1, and the injection volume was 1 μL. The oven temperature was held at 40°C for 1 min, and then raised to 100°C at 20°C·min^-1^, raised to 300°C at 15°C·min^-1^, and held at 300°C for 5 min. All samples were analyzed in scan mode. The ion source and transfer line temperature were 230°C and 280°C, respectively.

### 2.3 Statistical analysis

Excel 2017 software was used to perform preliminary data processing and calculate the mean. IBM SPSS Statistics 21.0 software was used for data T-text and correlation analysis. R version 4.2.3 software was used to produce box plots (R libraries was gghalves version 0.1.4), principal component analysis (PCA, R libraries was ggbiplot version 0.55), heat plots (R libraries was pheatmap 1.0.12), volcano plot analysis (R libraries was ggplot2 version 3.4.0), orthogonal partial least squares discriminant analysis (OPLS-DA, package used for this was ropls and mixOmics) (Jia et al., 2023), redundancy analysis (RDA, R libraries was vegan version 2.6.4) and correlation matrix analysis (R libraries was linkET 0.0.7.1) (Watkins, 2020). The entropy weight TOPSIS statistical analysis was performed on the SPSSAU online platform (https://spssau.com/) (Çelikbilek and Tüysüz, 2020).

## 3 Results and discussion

### 3.1 *Casuarina equisetifolia* rhizosphere soil characterization

#### 3.1.1 Physicochemical indexes

Continuous monoculture of plants may lead to changes in the physicochemical indexes of the rhizosphere soil, which in turn changes the nutrient content of the soil and affects plant growth (Guo et al., 2022; Wang et al., 2022). In this study, as the number of continuous planting of *C. equisetifolia* increased (M1-M3). (**Table 1**), the rhizosphere soil pH did not change significantly, while OM, CEC, TN, TP, TK, AN, AP and AK contents showed a decreasing trend.

**Table 1:** Analyses of physicochemical indexes of *Casuarina equisetifolia* rhizosphere soil collected after the first (M1), the second (M2) and the third (M2) continuous planting*.

Index	M1	M2	M3
pH value	5.34 ± 0.04a	5.38 ± 0.05a	5.31 ± 0.02a
Organic matter content (g·kg^-1^)	3.34 ± 0.03a	2.37 ± 0.07b	1.63 ± 0.02c
Cation exchange capacity (cmol·kg^-1^)	2.68 ± 0.07a	1.66 ± 0.05b	1.54 ± 0.03c
Total nitrogen (g·kg^-1^)	9.50 ± 0.07a	3.84 ± 0.05b	2.68 ± 0.08c
Total phosphorus (g·kg^-1^)	0.26 ± 0.02a	0.12 ± 0.01b	0.11 ± 0.02b
Total potassium (g·kg^-1^)	1.21 ± 0.02a	1.15 ± 0.01b	0.97 ± 0.02c
Available nitrogen (mg·kg^-1^)	23.130 ± 0.469a	7.60 ± 0.189b	6.29 ± 0.077c
Available phosphorus (mg·kg^-1^)	8.16 ± 0.05a	2.22 ± 0.02b	1.36 ± 0.06c
Available potassium (mg·kg^-1^)	105.89 ± 1.35a	95.58 ± 0.70b	86.98 ± 1.53c

* Data are the means of three replicates ± SE. For each physicochemical index, different letters indicate a significant level at p < 0.05.

#### 3.1.2 Microbial functional diversity

**Figure 1:**
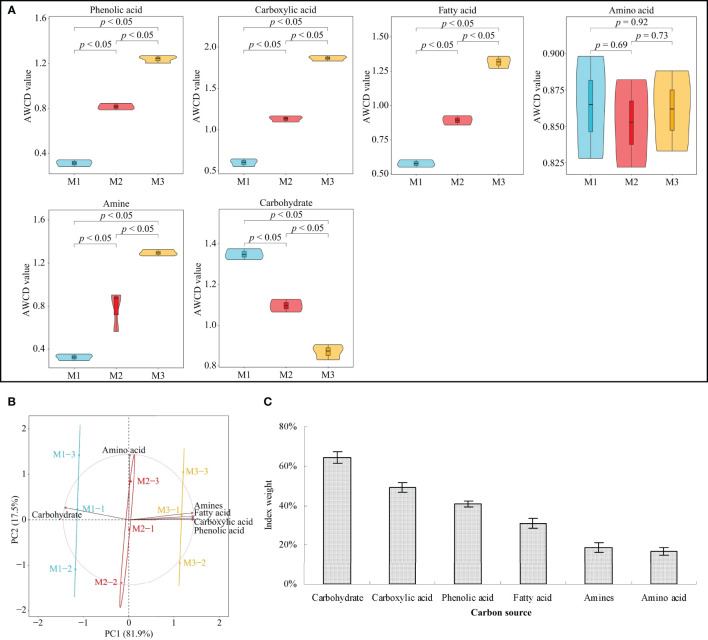
Functional diversity of *Casuarina equisetifolia* rhizosphere soil microorganisms determined by the BIOLOG ECO microplate method at the first (M1), second (M2) and third (M3) continuous planting expressed as utilization rate of carbon sources: **(A)** average well colour development (AWCD, data are the means of three replicates ± SE); **(B)** principal component analysis; **(C)** TOPSIS weighting (Data are the means of three replicates ± SE). Data are the means of three independent replicates.

Continuous planting imbalances soil microbial communities, alters the function of soil microbes, disturbs nutrient cycling in the soil, and, in turn, affects plant growth (Zhao et al., 2022). As BIOLOG ECO results (**Figure 1A**) showed, in the *C. equisetifolia* rhizosphere soil, the number of microorganisms utilizing phenolic acid, carboxylic acid, fatty acid, and amines as a carbon source significantly increases with the rise of continuous planting. At the same time, the number of microorganisms utilizing carbohydrates as a carbon source showed a significant decreasing trend, while no significant difference was recorded by microorganisms utilizing amino acid as a carbon source.

PCA (**Figure 1B**) effectively differentiated data on microbial communities collected at the three tested continuous planting conditions. Microorganisms using carbohydrates or amino acids as carbon sources were relevant in M1 and M2 samples, respectively. Microorganisms using phenolic acid, carboxylic acid, fatty acid, and amines as carbon sources characterized M3 samples. Starting with these preliminary remarks, TOPSIS weight (**Figure 1C**) revealed that continuous planting had the greatest effect on microorganisms using carbohydrate, carboxylic acid, or phenolic acid as carbon sources. This experimental evidence agrees with Xie et al. (2021) regarding the continuous cropping of peanuts. Continuous planting promotes the accumulation of carboxylic acid and phenolic acid in the rhizosphere soil, and the increment of microorganisms that use them as carbon sources. These groups of microorganisms include plant-pathogens which induce plant diseases or hamper plant growth (Li et al., 2021c). *C. equisetifolia* continuous planting reduces carbohydrates in rhizosphere soil and increases the concentration of fatty acid, amines, and especially carboxylic acid and phenolic acid in soil, which could alter the number and function of soil microorganisms and affect plant growth.

#### 3.1.3 Metabolomics

**Figure 2:**
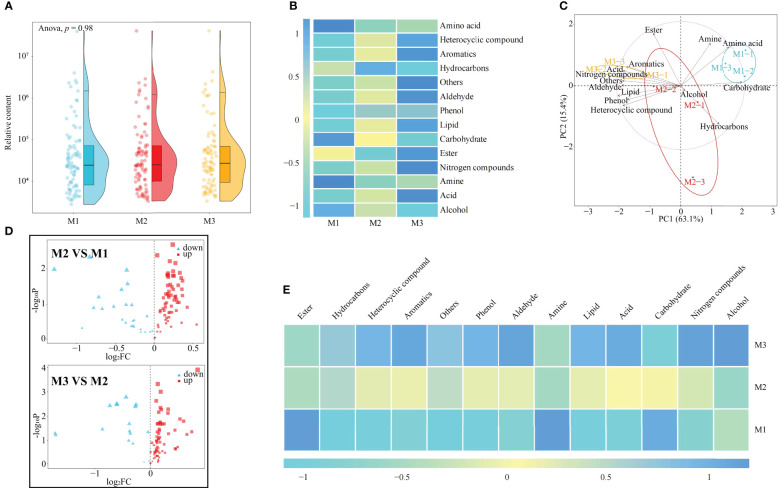
Effect of the first (M1), second (M2) and third (M2) continuous planting on *Casuarina equisetifolia* rhizosphere soil metabolomics: **(A)** metabolites relative content; **(B)** metabolite subdivision per chemical categories (color changes from -1 to 1 indicate small to large contents); **(C)** principal component analysis; **(D)** volcano plot analysis; **(E)** differential metabolites subdivided per chemical categories (color changes from -1 to 1 indicate small to large contents). Data are the means of three replicates. For metabolites definition and concentration see **Table S1**.

It has been reported that continuous planting of plants leads to changes in rhizosphere soil metabolites, which in turn affects plant growth (Pervaiz et al., 2020). In this study, it was found (**Figure 2A**; **Table S1**) that there was no significant difference in total rhizosphere soil metabolites among M1, M2 and M3 (*p* = 0.98). After classification of soil metabolites, their contents were analyzed (**Figure 2B**) and it was found that the content of acid, aldehyde, aromatics, heterocyclic compound, lipid, nitrogen compounds, phenol, and others tended to increase with the number of continuous plantings of *C. equisetifolia* (M1~M3), whereas the content of alcohol and carbohydrate tended to decrease. PCA (**Figure 2C**) effectively differentiated data on soil metabolites collected at the three tested continuous planting conditions. Soil metabolites such as aromatics, acid, nitrogen compounds, others, aldehyde, lipid, phenol, and heterocyclic were relevant in M1. Soil metabolites such as ester, alcohol, and hydrocarbons were relevant in M2. Soil metabolites such as amine, amino acid, and carbohydrate characterized M3 samples.

Accordingly, further analysis using volcano plots in this study revealed that the contents of 69 metabolites in the rhizosphere soil of *C. equisetifolia* changed significantly with the increase in the number of continuous planting, with 12 metabolites showing an increasing trend and 57 metabolites showing a decreasing trend (**Figure 2D**). The 69 metabolites could be categorized into 13 groups, of which 10 showed an increasing trend and 3 showed a decreasing trend as the number of continuous planting increased (**Figure 2E**).

**Figure 3:**
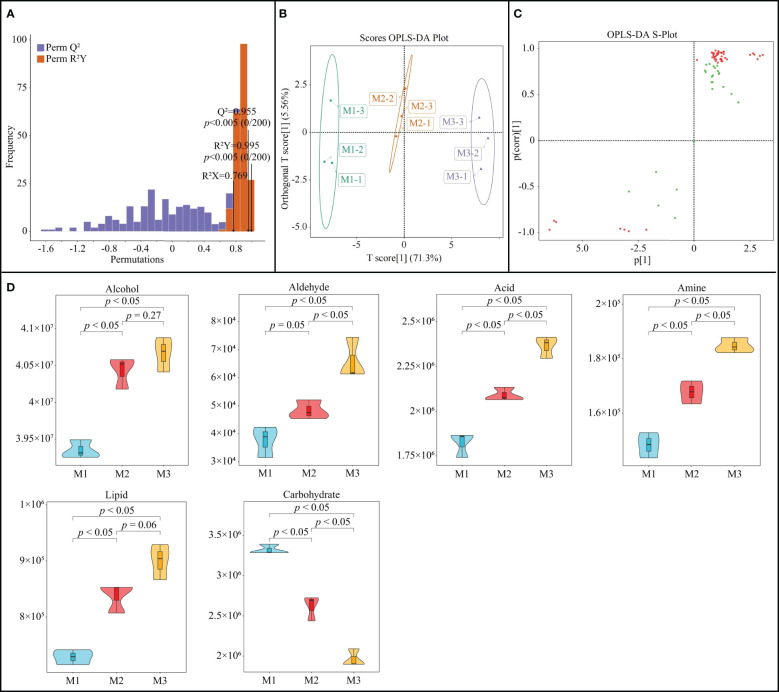
OPLS-DA model of the first (M1), second (M2) and third (M2) continuous planting on *Casuarina equisetifolia* rhizosphere soil key metabolites: **(A)** plot of the goodness-of-fit test; **(B)** differentiation analysis between and within sample groups; **(C)** distinction plot (red points indicate significantly different metabolites); **(D)** content analysis subdivided per chemical categories. Data are the means of three replicates.

The OPLS-DA model can be used to simulate the relationship of metabolites among different samples and to obtain metabolites with key differences through the variable importance projection values (VIP values) of different metabolites (Li et al., 2023). Moreover, after the model is constructed, the fit and predictability of the model need to be tested, and reaching a significant level indicates that the model is reasonably constructed before it can be used in subsequent analysis (Rivera-Pérez et al., 2022). On the basis of the previous differential metabolite analysis, this study further constructed the OPLS-DA model of rhizosphere soil metabolites of *C. equisetifolia* with different numbers of continuous planting. The results showed (**Figure 3A**) that the R^2^Y value of the goodness-of-fit of the OPLS-DA model was 0.995 after 200 random simulations and the Q^2^ value of predictability was 0.995, which were significant (*p* < 0.005). It can be seen that the model constructed in this study can effectively distinguish different samples and can be used for subsequent analysis. Scores OPLS-DA plot analysis showed (**Figure 3B**) that different samples could be effectively distinguished in different regions, with intra-group differences of 5.56% and inter-group differences of 71.3%. It can be seen that the reproducibility of three replicates of the same sample was high, while there were significant differences between samples. The S-Plot plot analysis of OPLS-DA showed (**Figure 3C**) that a total of 42 key soil metabolites were obtained, of which 35 metabolites showed an increasing trend and 7 metabolites showed a decreasing trend as the number of continuous planting increased.

Further classification analysis of the key metabolites showed (**Figure 3D**) that the 42 metabolites could be classified into six groups, of which the content of alcohol, aldehyde, acid, amine, and lipid showed a significant upward trend with the increase in the number of continuous planting, while the content of carbohydrate showed a significant decreasing trend. Alcohol can be oxidized to aldehyde, which is further oxidized to acid, and the content of alcohol and aldehyde in the soil rises, which in turn increases the accumulation of acid in the soil. It has been reported that continuous planting is highly likely to lead to an increase in the content of acids in the rhizosphere soil of plants, and the accumulation of large amounts of acids can inhibit plant growth (Bao et al., 2022). Cheng et al. (2020) found that the main substances that lead to the continuous planting obstacle of tomato belong to fatty acids, and reducing fatty acids in the soil can alleviate the phenomenon. Chen et al. (2023) found that continuous tobacco cropping leads to amines accumulation in rhizosphere soil, which in turn inhibits tobacco growth. Carbohydrate in soil is the main carbon source for microbial reproduction, which is closely related to microbial diversity and function, while continuous planting can lead to a decrease in its content, which in turn reduces soil microbial diversity and reduces soil nutrient cycling capacity (Wang et al., 2019; Xie et al., 2019). It can be seen that the continuous planting of *C. equisetifolia* leads to an increase in the content of acid, amine and lipid in the soil, which have an autotoxic effect, and an increase in the soil autotoxic effect; on the other hand, a decrease in the content of carbohydrate in the soil, which in turn may have reduced the diversity of soil microorganisms and reduced the nutrient cycling capacity of the soil.

### 3.2 Interaction network analysis

**Figure 4:**
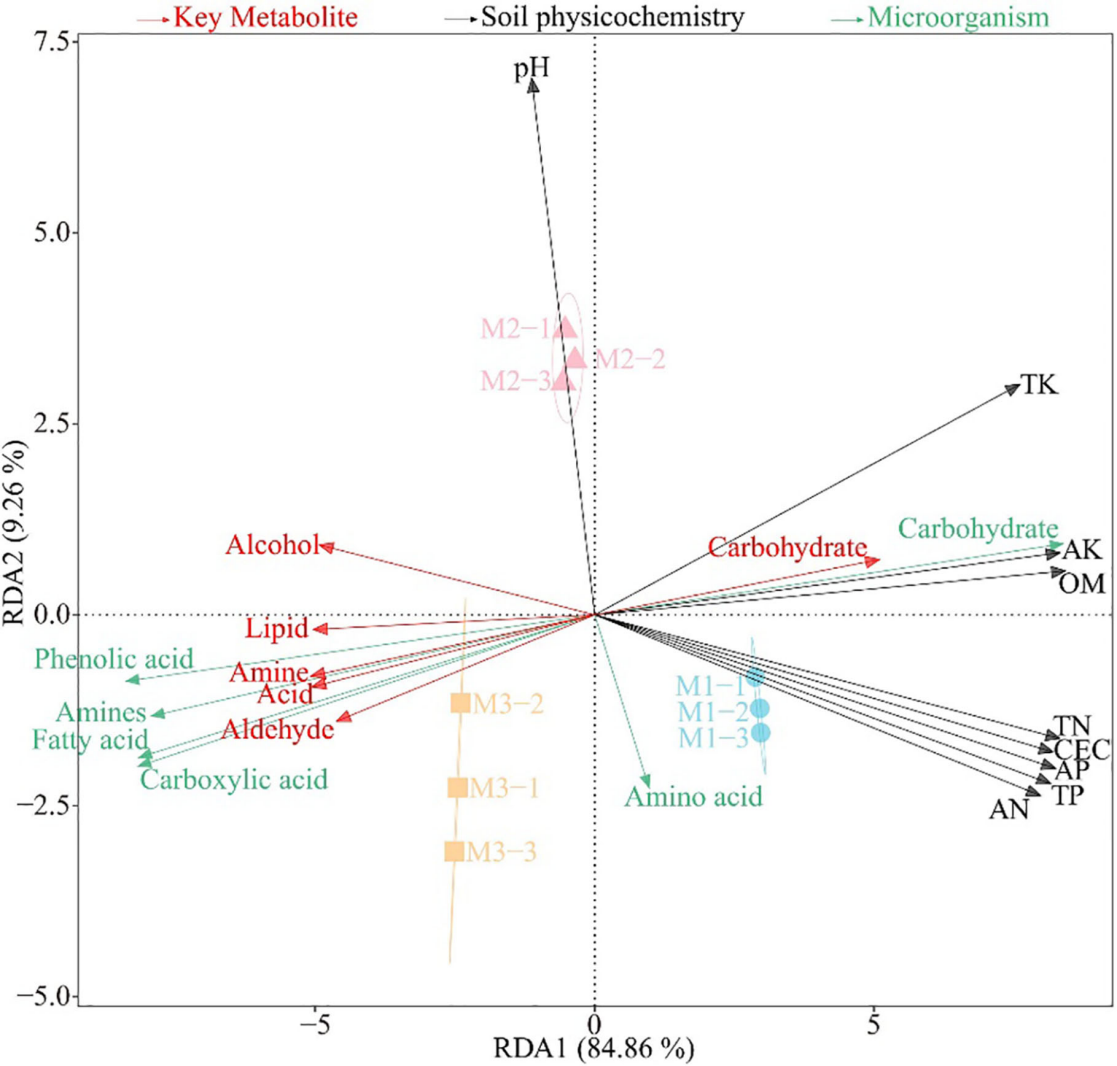
Redundancy analysis among physicochemical indexes (black words), microorganisms using different carbon sources (green words) and key metabolites (red words) in the rhizosphere soil after the first (M1), second (M2) and third (M2) continuous planting of *Casuarina equisetifolia*. Physicochemical indexes include organic matter content (OM), cation exchange capacity (CEC), total nitrogen (TN), total phosphorus (TP), total potassium (TK), available nitrogen (AN), available phosphorus (AP) and available potassium (AK). Average well colour development (AWCD) expresses microorganisms using different carbon sources.

On the basis of the previous study, this study further analyzed the interactions among physicochemical indexes, microorganisms and key metabolites in the rhizosphere soil of *C. equisetifolia*. The redundancy analysis (**Figure 4**) showed that the metabolite significantly associated with M1 was carbohydrate, the significantly associated microorganisms were microorganisms that used carbohydrate as a carbon source, and the significantly associated soil physicochemical indexes were TK, AK, OM, TN, CEC, AP, TP, and AN; and the metabolites significantly associated with M3 were alcohol, aldehyde, acid, amine, and lipid, and the significantly associated microorganisms were those using phenolic acid, carboxylic acid, fatty acid, and amines as carbon sources.

**Figure 5:**
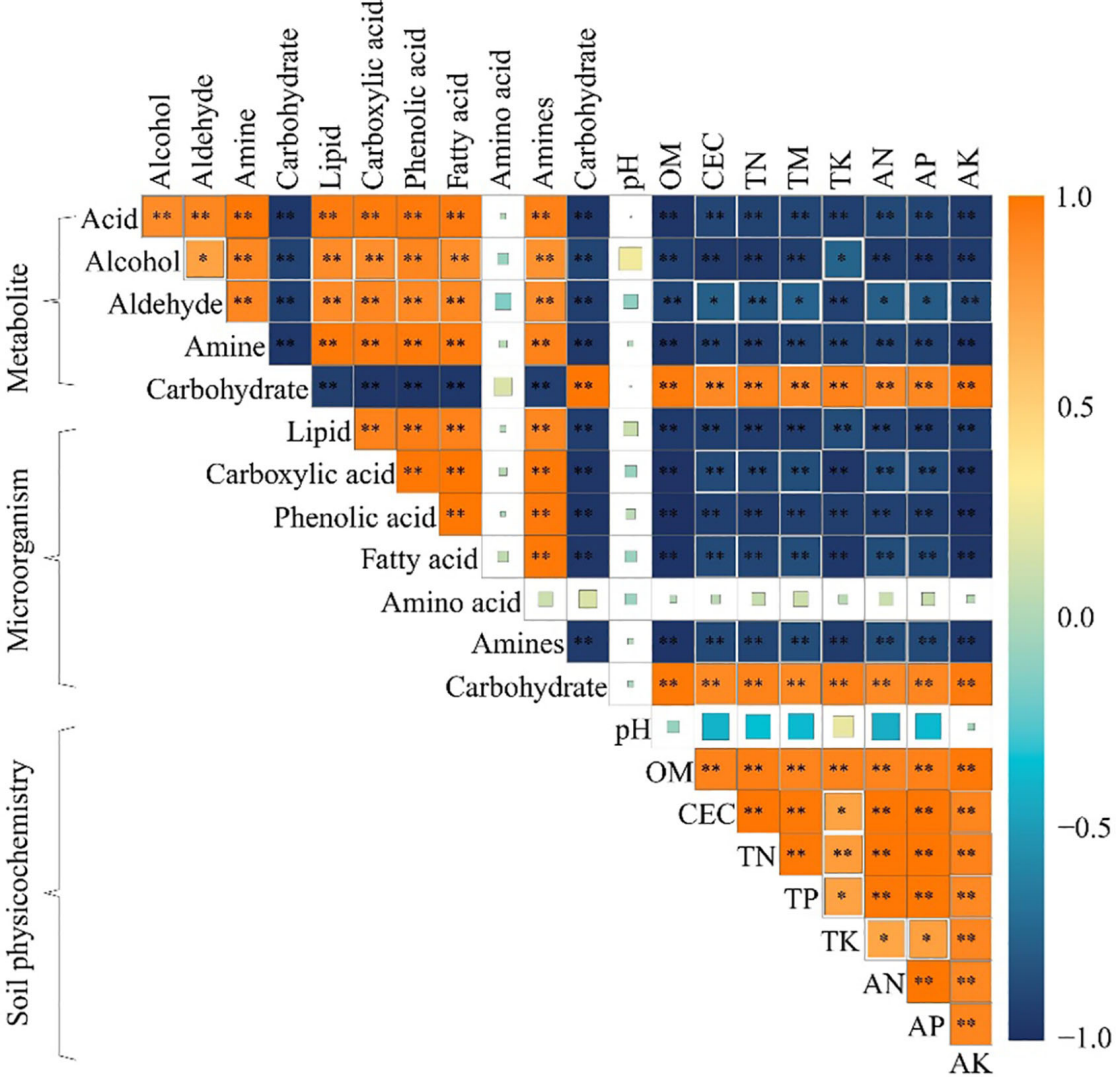
Correlation matrix heat map of physicochemical indexes, microorganisms using different carbon sources and key metabolites in the rhizosphere soil after the first (M1), second (M2) and third (M2) continuous planting of *Casuarina equisetifolia*. Physicochemical indexes include organic matter content (OM), cation exchange capacity (CEC), total nitrogen (TN), total phosphorus (TP), total potassium (TK), available nitrogen (AN), available phosphorus (AP) and available potassium (AK). Average well colour development (AWCD) expresses microorganisms using different carbon sources. The correlation ranges from +1 to −1. The variables were considered uncorrelated (0), positively (> 0) or negatively (< 0) correlated. “*” and “**” indicate statistical significance at *p* < 0.05 and *p* < 0.01 level, respectively.

The correlation matrix analysis (**Figure 5**) showed that rhizosphere soil key metabolites were not significantly correlated with soil pH and microorganisms using amino acid as carbon source; rhizosphere soil carbohydrate was significantly and positively correlated with microorganisms using carbohydrate as carbon source, and with soil physicochemical indexes, such as TK, AK, OM, TN, CEC, AP, TP and AN, whereas they were significantly and negatively correlated with microorganisms using phenolic acid, carboxylic acid, fatty acid, and amines as carbon sources. Secondly, alcohol, aldehyde, acid, amine and lipid in rhizosphere soil were significantly and negatively correlated with soil physicochemical indexes such as TK, AK, OM, TN, CEC, AP, TP, and AN, and significantly and positively correlated with microorganisms using phenolic acid, carboxylic acid, fatty acid, and amines as carbon sources.

Carbohydrate, alcohol, aldehyde, acid, amine and lipid are all important carbon sources for soil microbial propagation, and their levels affect the number of soil microorganisms that use them as carbon sources (Fallahi et al., 2021). It has been reported that the continuous planting of tea trees promotes the accumulation of acid and lipid in the soil, which in turn promotes the propagation of microorganisms that use phenolic acid, carboxylic acid, and fatty acid as carbon sources, and reduces the available nutrient content of the soil (Li et al., 2017). Continuous planting of tobacco promotes soil acid and amine accumulation, which in turn promotes the propagation of microorganisms using phenolic acid, carboxylic acid, and amines as carbon sources, and reduces soil nutrient cycling capacity (Yang et al., 2011). Continuous planting of *Rehmannia glutinosa* leads to a proliferation of soil microorganisms that use acid as a carbon source, which in turn leads to a decrease in available nutrients in the soil, and*Rehmannia glutinosa* growth is significantly inhibited (Wu et al., 2013). In summary, the content of different metabolites in rhizosphere soil changed significantly after continuous planting of *C. equisetifolia*, altering soil microbial quantity and function, reducing soil nutrient cycling capacity, and significantly reducing total nutrient and available nutrient content in rhizosphere soil.

## 4 Conclusion

**Figure 6:**
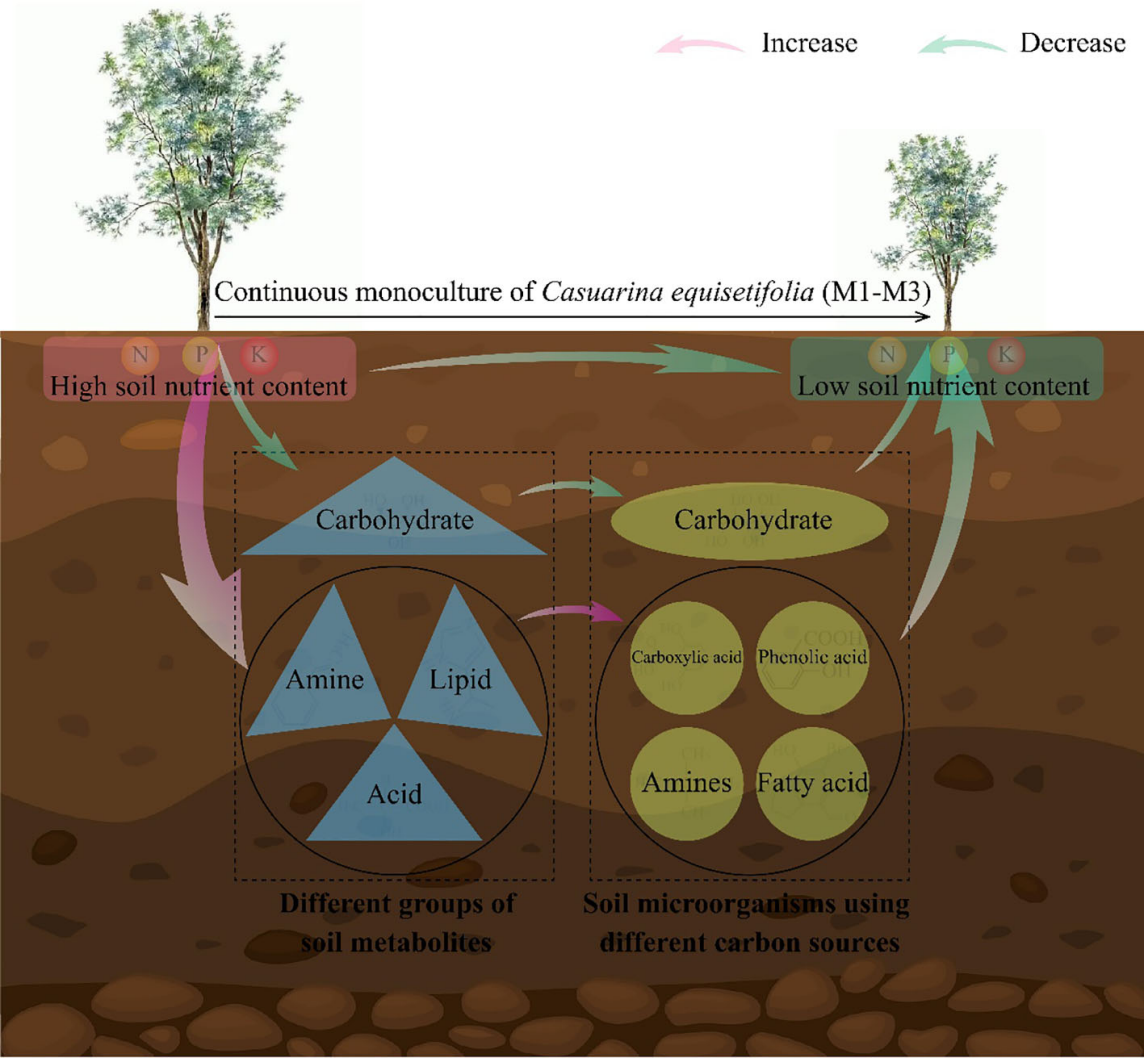
Mechanistic analysis of the effect of continuous planting on *Casuarina equisetifolia* rhizosphere soil physicochemical indexes, microorganisms and soil metabolomics. In the rhizosphere of *C. equisetifolia*, continuous monoculture: reduced carbohydrate concentration, soil microorganisms using carbohydrate as a carbon source, and nutrient cycling capacity; increased acid, amine and lipid content, and microorganisms using phenolic acid, carboxylic acid, fatty acid and amines as carbon sources; hindered *C. equisetifolia* plants growth.

In this study, it was found that continuous planting led to an increase in the levels of metabolites with autotoxic effects, such as acid, amine, and lipid, in the rhizosphere soil of *C. equisetifolia*, which, in turn, enhanced the propagation of microorganisms in the soil that use phenolic acid, carboxylic acid, fatty acid and amines as carbon sources. At the same time, continuous planting reduced carbohydrate content in the rhizosphere soil of *C. equisetifolia* and decreased the number of microorganisms in the soil that used carbohydrate as a carbon source. The results of the interactions network analysis showed that the rhizosphere soil physicochemical indexes of *C. equisetifolia* were significantly and positively correlated with carbohydrate content and the number of microorganisms using carbohydrate as a carbon source, while they were significantly and negatively correlated with the content of acid, amine, and lipid and the number of microorganisms using them as carbon sources. It can be seen (**Figure 6**) that continuous planting of *C. equisetifolia* reduced the rhizosphere soil carbohydrate content, increased the content of acid, amine, and lipid, and then reduced the number of soil microorganisms using carbohydrate as a carbon source, and increased the number of microorganisms using phenolic acid, carboxylic acid, fatty acid, amines as carbon sources, and finally reduced the nutrient cycling capacity of *C. equisetifolia.* In this study, we analyzed the causes leading to the formation of obstacle to continuous planting of *C. equisetifolia* from the perspectives of rhizosphere soil metabolites and microbial functional diversity, which is of great significance for the management of continuous planting of *C. equisetifolia* and soil remediation. However, changes in rhizosphere soil metabolite content of the continuous planting of *C. equisetifolia* affected microbial functioning, so are changes in microbial functioning related to changes in microbial communities? Which key microorganisms change significantly after continuous planting of *C. equisetifolia*, and is there some connection between them and soil nutrient cycling? Further research on this area needs to be explored in depth.

## Data availability statement

The datasets presented in this study can be found in online repositories. The names of the repository/repositories and accession number(s) can be found in the article/**Supplementary Material**.

## Author contributions

YW: Conceptualization, Formal Analysis, Methodology, Visualization, Writing – original draft, Writing – review & editing. JL: Conceptualization, Formal Analysis, Methodology, Visualization, Writing – original draft, Writing – review & editing. ML: Formal Analysis, Writing – original draft. XJ: Formal Analysis, Writing – original draft. YC: Formal Analysis, Writing – original draft. MH: Formal Analysis, Writing – original draft. QZ: Investigation, Methodology, Writing – original draft. PC: Investigation, Methodology, Writing – original draft. SL: Investigation, Methodology, Writing – original draft. WL: Funding acquisition, Resources, Writing – review & editing. HW: Conceptualization, Funding acquisition, Methodology, Project administration, Supervision, Visualization, Writing – original draft, Writing – review & editing. ZW: Conceptualization, Funding acquisition, Methodology, Project administration, Supervision, Visualization, Writing – original draft, Writing – review & editing.

## Supplementary material

Supplementary Table 1 Metabolites in rhizosphere soil of *Casuarina equisetifolia* with different numbers of continuous planting.
